# Molecular detection of respiratory pathogens and typing of human rhinovirus of adults hospitalized for exacerbation of asthma and chronic obstructive pulmonary disease

**DOI:** 10.1186/s12931-019-1181-0

**Published:** 2019-09-13

**Authors:** Fanny Wai-san Ko, Paul Kay-sheung Chan, Renee W. Y. Chan, Ka-Pang Chan, April Ip, Angela Kwok, Jenny Chun-li Ngai, So-Shan Ng, Chan Tat On, David Shu-cheong Hui

**Affiliations:** 1 SH Ho Research Center in Respiratory Diseases, Department of Medicine and Therapeutics, The Chinese University of Hong Kong, Hong Kong, People’s Republic of China; 2 Department of Microbiology, The Chinese University of Hong Kong, Hong Kong, People’s Republic of China; 3 Department of Paediatrics, The Chinese University of Hong Kong, Hong Kong, People’s Republic of China

**Keywords:** Viruses, Asthma exacerbations, COPD exacerbations

## Abstract

**Background:**

Acute exacerbations of chronic obstructive pulmonary disease (AECOPD) and asthma are associated with a variety of precipitating factors including infection. This study assessed the infective viral etiologies by real-time multiplex polymerase chain reaction of patients hospitalized with AECOPD and asthma exacerbations. In addition, infective etiologies were assessed for association with the clinical outcome of the patients.

**Methods:**

Adults admitted with AECOPD and asthma exacerbations between August 2016 and July 2017 were recruited. Nasopharyngeal aspirate (NPA) samples were obtained from the patients within 1–2 days of admission and subjected to pathogen detection and human rhinovirus (HRV) typing.

**Results:**

Altogether 402 patients with AECOPD, 80 stable COPD, 100 asthma exacerbation and 21 stable asthma subjects were recruited. Among those admitted for AECOPD and asthma exacerbations, 141(35.1%) and 45(45.0%) respectively had pathogens identified in the NPA specimens. The commonest virus identified was influenza A followed by HRV. HRV typing identified HRV-A and HRV-C as the more common HRV with a wide variety of genotypes. Identification of pathogens in NPA or HRV typing otherwise did not affect clinical outcomes including the hospital length of stay, readmission rates and mortality except that identification of pathogens in asthma exacerbation was associated with a lower rate of readmissions at 30 and 60 days.

**Conclusions:**

Many respiratory viruses were associated with AECOPD and asthma exacerbation. HRV-A and HRV-C were the more common HRV associated with exacerbations. Identification of pathogens in NPA was associated with less readmissions for asthma patients at 30 and 60 days.

**Trial registration:**

ClinicalTrials.gov NCT02866357.

**Supplementary information:**

**Supplementary information** accompanies this paper at10.1186/s12931-019-1181-0.

## Summary at a glance

For asthma and COPD exacerbations, identification of pathogens in nasopharyngeal aspirate or Human Rhinovirus (HRV) typing (most common HRV-A and HRV-C) did not affect clinical outcomes except that identification of pathogens in asthma exacerbation was associated with a lower rate of readmissions at 30 and 60 days.

## Background

Infectious agents are recognized as a major pathogenic factor in acute exacerbation of COPD AECOPD [1–3] and asthma. Other contributing factors for exacerbations include air pollution [4, 5]. low temperature, and interruption of regular treatment. Studies using polymerase chain reaction (PCR) reported variable detection rates of microorganisms between 22 and 60% in patients with AECOPD [6]. For asthma exacerbation, a previous study from the United Kingdom found that upper respiratory viral infections were associated with 80–85% of asthma exacerbations in children aged 9–11 years [7]. There are limited data on viral aetiologies for asthma exacerbation in adults. A recent study in Australia found that viral, bacterial and mixed aetiologies were identified in 32, 5 and 7% respectively of asthma exacerbations with hospital admissions [8].

HRVs are a major trigger of AECOPD and asthma exacerbations and may pose a significant problem for disease management [7, 9, 10]. HRV is the most common virus found in AECOPD with some studies reporting up to 69% among all the viral isolates [6]. Among the respiratory viruses, HRV infection preceded as many as 50% of asthma exacerbations in children [11]. Over 100 types of HRVs have been characterized and classified into 3 groups: HRV-A, HRV-B, and more recently HRV-C [12]. With advances in technology, it is now possible to identify different groups of HRV [13]. To the best of our knowledge, there are no data on the types of HRV that may precipitate AECOPD. There are very limited data on the subtypes of HRV that is related to adults with asthma exacerbations [14]. Furthermore it is unknown that if certain viral infections would be associated with worse clinical outcomes of the patients, such as prolonged hospital length of stay (LOS) and readmissions.

The primary aim of this study was to evaluate the prevalence of different viruses (including the typing of HRV) in relation to acute exacerbations of asthma and COPD. The secondary aim was to assess if certain pathogens had associations with clinical outcomes including duration of hospitalization, 30 and 60-day readmissions and mortality.

## Methods

Subjects with asthma exacerbation or AECOPD who were admitted to the Prince of Wales Hospital during 1 Aug 2016 and 31 July 2017 were screened for this study. AECOPD was defined when a patient with age ≥ 40 years and background COPD (lung function parameters of FEV_1_/FVC ratio < 70%) [15] presented with at least two of the following major symptoms (increased dyspnoea, increased sputum purulence, increased sputum volume) or one major and one minor symptom (nasal discharge/congestion, wheeze, sore throat, cough) for at least two consecutive days [16, 17]. Patients with asthma exacerbations were subjects aged ≥18 years, who had background asthma (with a consistent history, prior documented evidence of variable airflow obstruction and evidence of an increase in FEV_1_ greater than 12% and 200 mL following bronchodilator or bronchial hyperresponsiveness on bronchial provocation testing, when stable) [3], presented with either increasing dyspnea, wheeze or cough for at least 2 consecutive days.

Control asthma and COPD subjects were recruited from the respiratory clinic of the Prince of Wales Hospital. All recruited COPD subjects should have an FEV_1_/FVC ratio of < 70%. For asthma patients, they should have a clinical diagnosis of asthma (as above). These subjects should have no exacerbation of asthma or COPD for 6 weeks prior to the assessment.

Patients with a history of lung resection or other significant pulmonary diseases such as pulmonary fibrosis, active infection such as pulmonary tuberculosis and having short life expectancies including subjects with terminal malignancy or intractable heart failure were excluded from this study.

After obtaining consent from the patient, nasopharyngeal aspirate (NPA) was obtained from the patients within 1–2 days of hospitalization for exacerbation of asthma and COPD. This study was approved by the Joint CUHK-NTEC Clinical Research Ethics Committee (CRE-2015.164).

The NPA specimens collected were put immediately in viral transport medium and kept at 4 °C during transportation. Both viral RNA and DNA were extracted from the specimens using QIAamp® MinElute® Virus Spin Kit (Qiagen, Germany) and subjected to compliance with the European Union In-Vitro Diagnostics requirement. 22 respiratory pathogens (coronavirus 229E, HKU1, NL63, OC43, human metapneumovirus, influenza A (including H1 and H3), influenza A H1N1pdm09, influenza B, parainfluenza 1, 2, 3 & 4, rhinovirus/enterovirus, RSV-A and RSV-B, adenovirus, bocavirus, *Bordetella pertussis, Chlamydophila pneumoniae, Legionella pneumoniae,* and *Mycoplasma pneumoniae*)*.* were assessed by RespiFinder® 2SMART (PathoFinder, Netherlands) kit according to the manufacturer’s instructions. Real-time multiplex PCR was performed on a Rotor-Gene Q® MDx instrument (Qiagen, Germany) and detection was based on melting curve analysis. Specimens positive for HRV/enterovirus were subjected to further sequencing work for virus typing, adopted from the method we described in our previous study [13].

Subjects were called back to our research clinic 6–8 weeks after discharge for lung function assessment. Spirometry pre and post-bronchodilator according to the American Thoracic Society and European Respiratory Society standards was performed [18]. The updated predicted spirometry values for Hong Kong Chinese was used to calculate the predicted lung function [19]. The subsequent hospital admissions and mortality were recorded after the baseline assessment for up to 2 months.

For control COPD and asthma patients, we identified patients from the out-patient clinic and called back these patients for assessment on a single visit for obtaining NPA. Their spirometry was tested in the same visit.

We aimed at recruiting 400 episodes of AECOPD, 100 episodes of asthma exacerbation and 1/5 the number of exacerbation of subjects as controls. Given the potential seasonal variation in viral etiologies, we used the time-stratified random sampling approach for patient recruitment (each month we recruited a similar number of subjects in different categories).

Data were analyzed by the Statistical Package of the Social Science Statistical software (SPSS) for Window, Version 22.0.0 (IBM SPSS Inc., IL, USA). Descriptive statistics were applied to the prevalence of different viruses found in AECOPD and asthma patients and comparisons with the control subjects were done by chi-square test, Fisher’s exact test and Student t-test as appropriate. Clinical outcomes of the patients with different viruses were compared by chi-square test, Student t-test and Kruskal–Wallis test as appropriate. Figures were presented as mean (SD) or median (interquartile range), and a *p*-value of < 0.05 was considered as significant.

## Results

Between 1 Aug 2016 and 31 Jul 2017, we recruited 402 patients with AECOPD, 80 stable COPD, 100 with asthma exacerbation, and 21 with stable asthma subjects for this study. The characteristics of the COPD and asthma subjects are shown in Table 1. The exacerbation and stable state subjects were matched for lung function but not age, gender and inhaler therapies.

**Table 1:** Characteristics of the COPD and asthma patients

	Subjects with acute exacerbation of COPD (*n* = 402)	Control COPD (*n* = 80)	*p*-value (compared acute exacerbation and stable COPD)	Subjects with asthma exacerbation of (*n* = 100)	Control asthma (*n* = 21)	p-value (compared acute and stable asthma)
Gender (Male)	367 (91.3%)	78 (97.5%)	0.03*	34 (34.0%)	12 (57.1%)	0.04*
Age^a^	77.4 ± 8.6	73.2 ± 7.4	0.00*	64.8 ± 17.7	48.0 ± 14.6	0.00*
Influenza vaccination in the last 12 months	150 (37.3%)	33 (41.2%)	0.50	26 (26.0%)	2 (9.5%)	0.15
Types of residence			0.00*			0.12
Live alone	39 (9.7%)	4 (5.0%)		11 (11.0%)	0 (0.0%)	
Old age home resident	42 (10.4%)	0 (0.0%)		3 (3.0%)	0 (0.0%)	
Live with family	321 (79.9%)	76 (95.0%)		86 (86.0%)	21 (100.0%)	
Current smokers	68 (16.9%)	18 (22.5%)	0.20	4 (4.0%)	2 (9.5%)	0.291
Medications before admission						
any ICS	317 (78.9%)	68 (85.0%)	0.14	76 (76.0%)	19 (90.5%)	0.12
ICS with LABA	286 (71.1%)	66 (82.5%)	0.02*	65 (65.0%)	18 (85.7%)	0.05*
LABA+LAMA	199 (49.5%)	75 (93.8%)	< 0.001*	30 (30.0%)	4 (19.0%)	0.23
Comorbidities						
Old tuberculosis	67 (16.6%)	9 (11.3%)	0.22	4 (4.0%)	0 (0.0%)	1.00
Bronchiectasis	21 (5.2%)	2 (2.5%)	0.39	4 (4.0%)	0 (0.0%)	1.00
Congestive heart Failure	36 (8.9%)	4 (5.0%)	0.24	9 (9.0%)	0 (0.0%)	0.35
Ischaemic heart disease	38 (9.4%)	5 (6.2%)	0.35	7 (7.0%)	1 (4.7%)	1.00
Hypertension	211 (52.4%)	29 (36.2%)	0.00*	39 (39.0%)	4 (19%)	0.08
Hyperlipidaemia	65 (16.1%)	9 (11.2%)	0.26	17 (17.0%)	1 (4.7%)	0.19
DM	79 (19.6%)	12 (15.0%)	0.33	19 (19.0%)	2 (9.5%)	0.52
Osteoarthritis	8 (1.9%)	2 (2.5%)	0.67	4 (4.0%)	0 (0.0%)	1.00
Lung function						
Post-brochodilator FEV % predicted (%)^a^	45.8 ± 20.5	46.7 ± 17.6	0.71	73.8 ± 20.2	80.3 ± 22.6	0.23
Post-brochodilator FEV1/FVC ratio (%)^a^	50.4 ± 15.5	47.1 ± 12.9	0.08	69.1 ± 11.6	74.9 ± 11.8	0.06
During Admission						
Symptoms						
Cough	347 (86.3%)			81 (81.0%)		
Wheeze	192 (47.8%)			62 (62.0%)		
Fever	133 (33.1%)			32 (32.0%)		
Runny nose	15 (3.7%)			9 (9.0%)		
SOB	380 (94.5%)			88 (88.0%)		
Sputum production	344 (85.6%)			74 (74.0%)		
Sore throat	31 (7.7%)			18 (18.0%)		
Chest pain	48 (11.9%)			9 (9.0%)		
Hemoptysis	6 (1.5%)			1 (1.0%)		
Use of Non-invasive positive pressure ventilation	70 (17.4%)			5 (5.0%)		
Use of invasive mechanical ventilation	1 (0.2%)			0 (0.0%)		
Intensive care admission	2 (0.5%)			1 (1.0%)		

Data are presented as mean ± SD^a^ or number (percentages)

*ICS* inhaled corticosteroid, *LABA* long-acting beta-agonist, *LAMA* long-acting anti-muscurinic agent

* *p* < 0.05

**Fig. 1:**
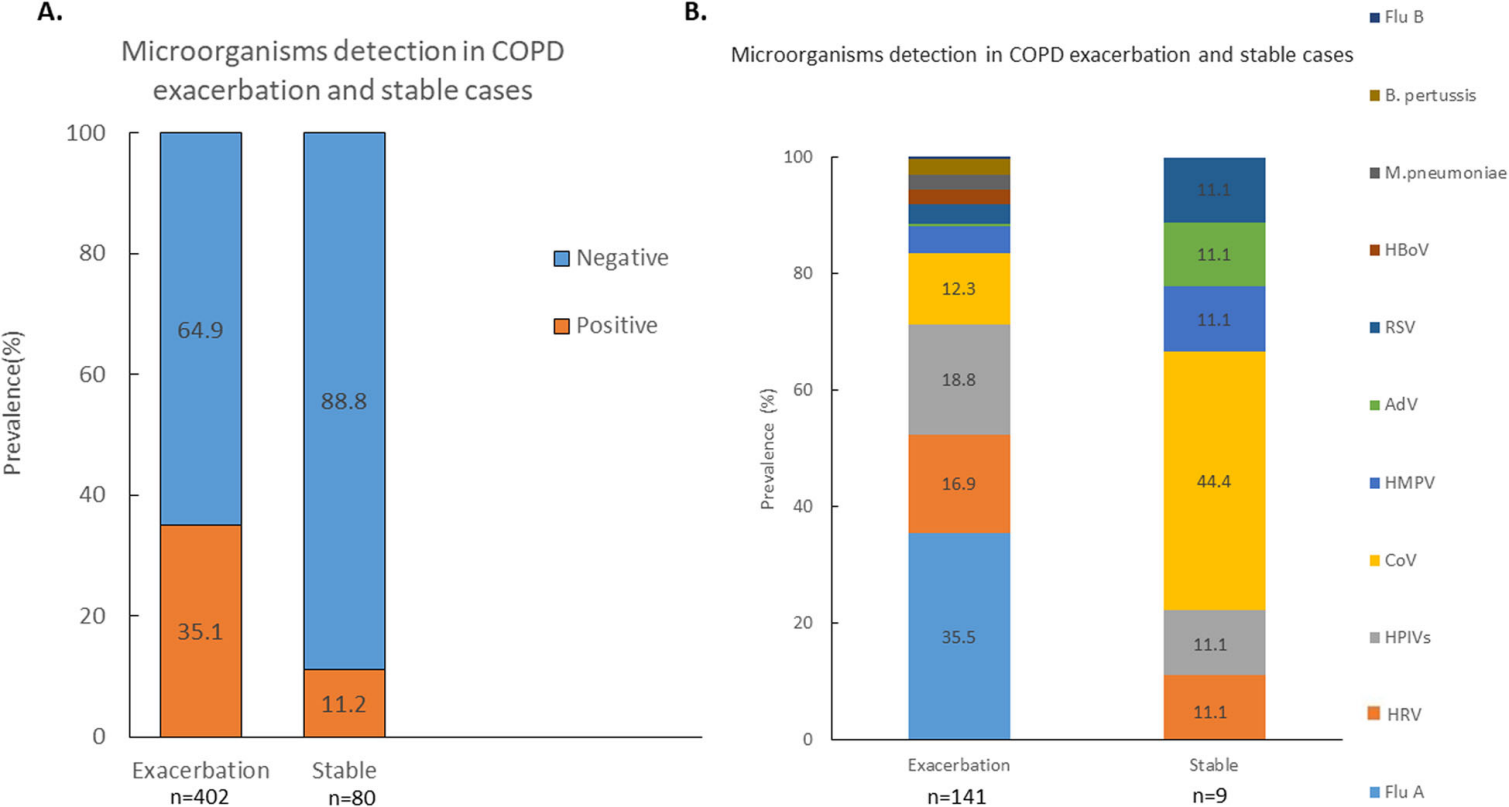
NPA results of the COPD cases. **a**: Patients with acute exacerbation of COPD and stable COPD with or without microorganisms detected in the NPA. **b**: Percentages of individual microorganism found in NPA in the subjects. ADV = adenovirus; B. Pertussis = *Bordetella pertussis;* CoV=Coronavirus; *Flu* A = influenza A; Flu B = influenza B; HBoV = Human Bocavirus; HMPV = *Human metapneumovirus; HPIV=Human parainfluenza virus;* M pneumoniae = *Mycoplasma pneumoniae;* HRVs = Human rhinovirus; RSV = Respiratory syncytial virus

Among the 402 subjects with AECOPD, 141(35.1%) cases had pathogens detected. 13 cases (3.2%) had co-infections with more than 1 pathogen identified. Among the 80 COPD control subjects, 9(11.2%) patients had pathogens detected. None had more than 1 pathogen identified in the NPA. The prevalence of microorganisms identified in the COPD (AECOPD and stable) patients is shown in Fig. 1. The prevalence of microorganisms identified in different months is shown in Additional file 1: Figure S1. Only the AECOPD group and asthma exacerbation group (not control subjects), had influenza A identified by RTPCR in the NPA. For those with AECOPD and asthma exacerbation subjects, 5 out of the 50 (10.0%) and 1 out of 19 subjects (5.26%) had the H1N1pdm09 strain identified respectively. The rest of the influenza A belonged to the H3N2 strain.

For the AECOPD subjects, those with pathogens identified in the NPA had similar hospital LOS (11.7 ± 9.6 vs 12.9 ± 10.5 days, *p* = 0.29), 30 day readmissions (0.23 ± 0.50 vs 0.28 ± 0.52times, *p* = 0.35), 60 day readmissions (0.39 ± 0.66 vs 0.53 ± 0.88times, *p* = 0.06), in-hospital mortality (2.8 vs 3.1%, *p* = 0.59), 30 day mortality (5.7 vs 5.8%, *p* = 0.58) and 60 day mortality (8.5 vs 8.1%, *p* = 0.51) compared with those without isolation of pathogens.

**Fig. 2:**
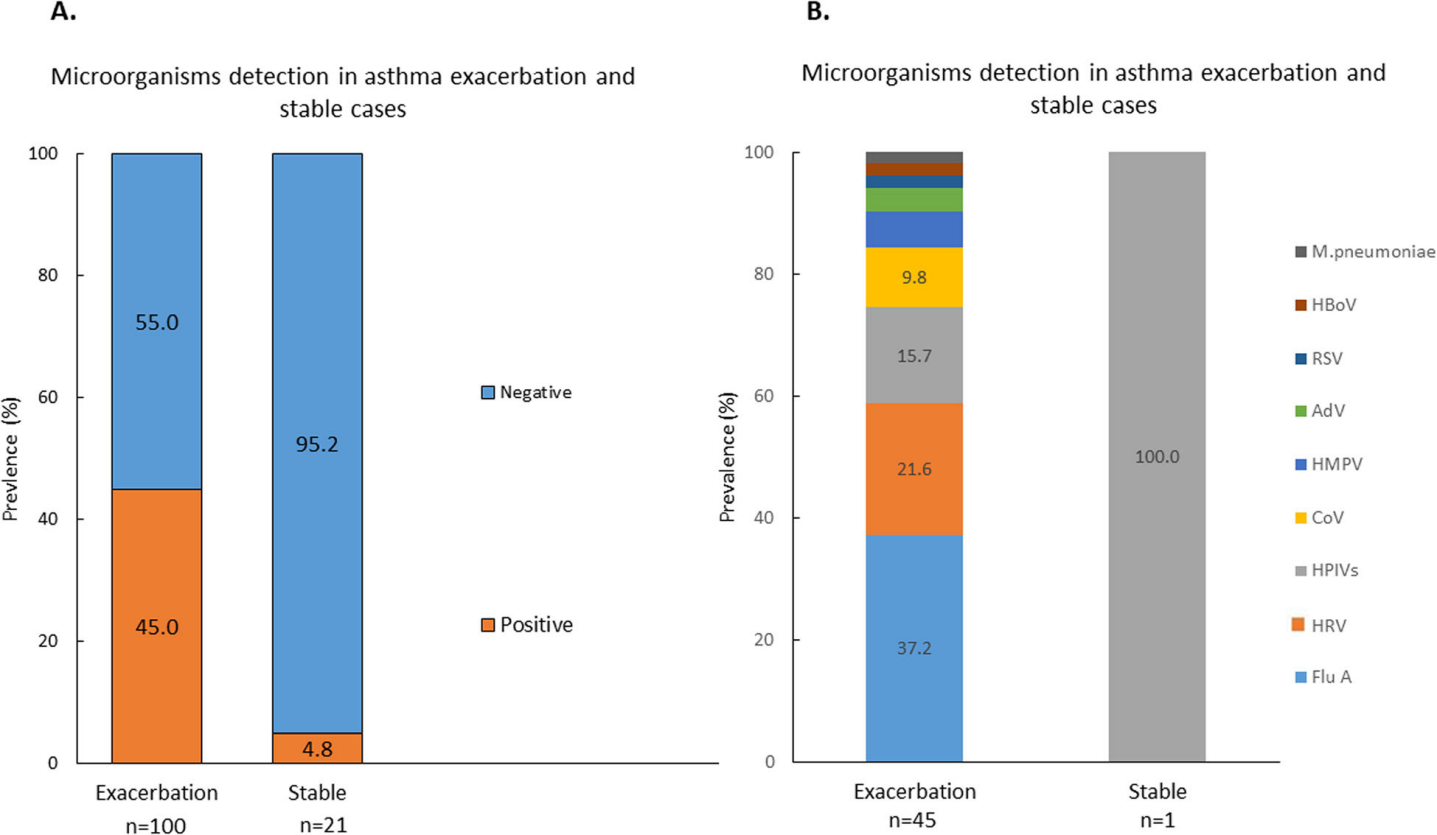
NPA results of the asthma cases. **a**: Patients with acute exacerbation of asthma and stable asthma with or without microorganisms detected in the NPA. **b**: Percentages of individual microorganism found in NPA in the subjects. ADV = adenovirus*;* CoV=Coronavirus; *Flu* A = influenza A; HMPV = *Human metapneumovirus; HPIV=Human parainfluenza virus;* M pneumoniae = *Mycoplasma pneumoniae*; HRVs = Human rhinovirus; RSV = Respiratory syncytial virus

Among 100 subjects with asthma exacerbation, 45(45.0%) had pathogens detected. 5(5.0%) subjects had co-infections with more than 1 pathogen studied. Among the 21 asthma control subjects, only 1(4.8%) subject had parainfluenza virus detected. The prevalence of microorganisms identified in asthma (exacerbated asthma and stable) patients is shown in Fig. 2. The prevalence of microorganisms identified in different months is shown in Additional file 1: Figure S2.

Subjects with asthma exacerbations with detection of pathogens in the NPA had similar hospital LOS (8.4 ± 6.8 vs 9.0 ± 12.2 days, *p* = 0.80) and in-hospital mortality (0 vs 1.8%, *p* = 0.55) in comparisons with those without pathogens. There was no mortality recorded after discharge of patients till 60 days. The NPA positive group had lower 30 day (0.04 ± 0.21 vs 0.35 ± 0.67times, *p* = 0.003) and 60 day readmissions (0.11 ± 0.32 vs 0.38 ± 0.73times, *p* = 0.02) than NPA negative group.

There was no difference in the rates of ICU admission, non-invasive ventilation (NIV) use and mechanical ventilation support among those with microorganisms identified in the NPA vs those without microorganisms identified for both asthma and COPD exacerbations. Results are shown in Table 2.

**Table 2:** Rate of intensive care unit admission and ventilation in relation to microorganisms identification

	COPD exacerbation cases		
	+ ve viruses (*n* = 141)	-ve viruses (*n* = 261)	*P*-value
ICU admission	1 (0.7%)	1 (0.4%)	1.00
NIV use	21 (14.9%)	49 (18.8%)	0.32
Mechanical Ventilation	1 (0.7%)	0 (0.0%)	0.35
	Asthma exacerbation cases		
	+ ve viruses (*n* = 45)	-ve viruses (*n* = 55)	*P*-value
ICU admission	1 (2.2%)	0 (0.0%)	0.45
NIV use	4 (7.2%)	1 (2.2%)	0.37
Mechanical Ventilation	0 (0.0%)	0 (0.0%)	NA

*ICU* intensive care unit, *NIV* non-invasive ventilation

Comparison of subjects with and without influenza vaccination found that the rate of influenza-related exacerbations appeared to be higher in those with influenza vaccination but that did not reach statistical significance. Results are shown in Table 3.

**Table 3:** Subjects with and without influenza vaccination and rate of admission for exacerbations with influenza A or B isolated in the NPA among subjects with COPD and asthma

	AECOPD (*n* = 402)	Asthma exacerbation (*n* = 100)
With flu vaccination	150 / 402	26 / 100
No. with Flu A or Flu B in subjects with flu vaccination	26/ 150, (17.3%)*	6 / 26, (23.1%)#
Without flu vaccination	252 / 402	74 /100
No. with Flu A or Flu B in subjects without flu vaccination	29 /252, (11.5%)*	13 / 74, (17.6%)#

Comparing those with Influenza A or B identified in NPA among the subjects with or without influenza vaccination in the COPD* and asthma# acute exacerbation subjects:*p = 0.133; #*p* = 0.57

HRV was detected in 26, 11 and 1 subjects respectively with AECOPD, asthma exacerbations and stable COPD. No subject in stable asthma group had HRV identified in the NPA. The subtypes are listed in Table 4.

**Table 4:** Types of rhinoviruses identified in the subjects

	AECOPD	Stable COPD	Asthma exacerbation	Stable asthma
HRV_A	HRV_A1 HRV_A7 HRV_A21 HRV_A33 HRV_A33 HRV_A36 HRV_A36 HRV_A43 HRV_A47 HRV_A49 HRV_A54 HRV_A68 HRV_A68 HRV_A75 HRV_A96	None	HRV_A21 HRV_A24 HRV_A34 HRV_A44 HRV_A73 HRV_A88	None
HRV_B	HRV_B37 HRV_B37 HRV_B99	None	HRV_B14	None
HRV_C	HRV_C1 HRV_C7 HRV_C15 HRV_C17 HRV_C19 HRV_C35 HRV_C45 HRV_C49	HRV_C12	HRV_C11 HRV_C18 HRV_C35 HRV_C45	None

Comparisons of different types of HRV (HRV-A, HRV-B and HRV-C) identified found that there was no association between the various types of HRV in both AECOPD and asthma exacerbations with hospital LOS, readmission and mortality. The results of the relationships of HRV types and clinical outcomes of AECOPD and asthma exacerbation are shown in Additional file 1: Tables S1 and S2 respectively.

## Discussion

Using multiplex molecular detection of respiratory pathogens of adults hospitalized for AECOPD and asthma exacerbations, this study has found that the rates of pathogens identification in the NPA were 35 and 45% respectively. The rates were much lower among the corresponding control COPD and asthma subjects, which were 11.2 and 4.8% respectively. HRV typing suggested that HRV-A and HRV-C were the more common HRV that were associated with AECOPD and asthma exacerbations with a wide variety of genotypes. Detection of microorganism in NPA or HRV typing otherwise did not affect the clinical outcomes including the hospital LOS, readmissions and mortality except that patients with asthma exacerbation, identification of pathogens was associated with a lower rate of readmissions at 30 and 60 days.

With advances in diagnostic technology, the rate of pathogen identification is higher in the current study than our previous study 10 years ago (35.1 vs 22%) [1], and influenza A viruses remained the most common pathogen identified. Most studies reported that HRV was the commonest virus identified [6]. In contrast, relatively few studies reported influenza A as the most frequently found virus [20, 21].

Among the asthma subjects, 45.0% had pathogens detected in this study. Most of the previous studies on detection of respiratory pathogens in asthma exacerbations were conducted in children [22]. A Korean study found that in adult asthma subjects with acute exacerbation, 26.3% had viruses identified in expectorated sputum, with HRV being the commonest virus detected [23]. A paediatric study in Hong Kong with children aged 3–18 years between January 2007 and February 2008 detected respiratory pathogens in 51.0% subjects, with HRV being the commonest organisms identified [24]. Adult subjects with asthma exacerbation thus appeared to have a lower rate of pathogens identified than in children.

Seasonal variations in the microorganisms identified in the NPA were observed in this study with influenza viruses reaching the peak in November to February and June to July for AECOPD. A similar pattern was observed for adult asthma exacerbation with influenza virus detection which peaked in November and June to July. This pattern was in phase with the influenza seasonal patterns in Hong Kong [25]. A study of HRV related exacerbation of airway diseases in the UK were related to childhood asthma with a seasonal increase of HRV infections in September through December and again in Spring [26]. Similar patterns were noted for adult COPD and asthma exacerbation patients in this study.

For children, HRV-A and HRV-C are more frequently associated with asthma exacerbations. A cross-sectional study of subjects aged < 18 years in Hong Kong between October 2008 and March 2009 found that asthma exacerbation was strongly associated with HRV and specifically HRV-C infections [13]. Similar observation for asthma children in other parts of the world was observed [27–29]. HRV-C appeared more strongly associated with more severe exacerbations, including those requiring hospitalization [30, 31]. In contrast, our adults with asthma exacerbation had HRV-A being isolated from the NPA more frequently than HRV-C. The pattern thus appeared different from the findings in children. A study in Japan showed that HRV-A or HRV-C had a potential role in precipitating asthma attacks among Japanese adult patients, with a broad genetic divergence [14].

Data on the type of HRV that are associated with AECOPD are scarce. There are more than 168 distinct HRV genotypes, including 80 HRV-A, 32 HRV-B serotypes and 65 newly identified HRV-C genotypes. The HRV structural and genetic variability has inhibited efforts to develop antivirals [14]. Studies in COPD patients generally have not typed the HRV.

Those with history of influenza vaccination appeared to have a higher rate of identification of influenza viruses in the NPA than those without vaccination for both AECOPD and asthma exacerbation though this did not reach statistical significance. A previous study suggested that protective serum antibody titres in COPD patients after influenza vaccination was lower than control subjects [32]. Overall the mortality benefit of influenza vaccine in COPD is suggested by some studies but the impact on exacerbation prevention is less clear [33]. Another systemic review indicated a favorable benefit-risk ratio for seasonal influenza vaccination in patients with COPD [34]. For persons with asthma, a recent systematic review observed that influenza vaccination might be effective in both reducing influenza infection and asthma attacks [35]. However, another Cochrane review has expressed uncertainty of the degree of protection that influenza vaccination might confer against asthma exacerbations that are related to influenza infection [36]. During the study period from August 2016 to July 2017, influenza A(H3N2) was the main circulating strain causing the Summer and Winter epidemics in Hong Kong [37]. Influenza vaccine effectiveness depends on many factors including matching between vaccine strains and the circulating strains, immunocompetence, age, repeated vaccination, antigenic sites and distances in addition to egg adaptation especially for influenza A(H3N2) virus [38]. A previous systematic review found substantial variations in influenza vaccine effectiveness across types and subtypes of influenza with substantially lower protection against H3N2 [39]. Examination of influenza vaccine effectiveness by serology is beyond the scope of this study as it would require a large sample size. In addition, as these patients had some time delay in presenting to the hospitals, it would be difficult to determine whether this was due to current or previous infection or vaccination by measuring influenza antibody based on haemagglutinin inhibition. While identification of pathogens in the NPA was associated with a lower rate of readmissions for asthma, exacerbations of COPD and asthma might also be due to other acute and transient factors such as exposure to air pollutants [4, 5], change in temperature, etc.

One of the limitations of this study is that this was a single-centre study. As the study mainly aimed to assess the subjects in exacerbation state, only 1/5 the number of exacerbation of subjects were recruited as controls. Furthermore, the same subjects were not tested between exacerbations due to the limitation of budget. The exacerbation and stable state subjects were not matched for gender, age and inhaler therapies. There were other factors that might be associated with AECOPD and asthma exacerbations, e.g. air pollution, weather changes, allergic sensitization, etc. that were not investigated. Bacterial infection might also play a part in AECOPD and this was not assessed in this study. Interaction of the microorganisms with other factors such as air pollutions and weather may be important. Although viral and atypical bacteria were identified in some subjects with exacerbations, we could not establish a causative role as some stable subjects also had pathogens identified in their NPA. The subjects involved in this study had severe airway disease exacerbations requiring hospitalizations and the result could not be extrapolated to those with milder illnesses being managed in the community.

## Conclusions

The results in this study provided the latest pattern of viral and atypical pathogens that were associated with AECOPD and asthma exacerbations in hospitalized patients. HRV typing suggested that HRV-A and HRV-C were the more common HRV subtypes that were associated with AECOPD and asthma exacerbations with a wide variety of genotypes. Presence of microorganisms in the NPA or HRV typing otherwise did not affect the clinical outcomes including the hospital LOS, readmissions and mortality except that patients who had infective aetiology identified during asthma exacerbation had lower rates of readmissions at 30 and 60 days.

## Supplementary information

**Additional file 1: Table S1.** Length of hospital stay, readmissions and mortality in relations to types of human rhinovirus identified in the NPA in patients with acute exacerbation of COPD. **Table S2.** Length of hospital stay, readmissions and mortality in relations to types of human rhinovirus identified in the NPA in patients with acute exacerbation of asthma. **Figure S1.** NPA results of the COPD exacerbation cases in different months. **Figure S2.** NPA results of the asthma exacerbation cases in different months. (DOCX 145 kb)

## Data Availability

Data sharing can be provided to researchers who provide a methodologically sound proposal.

## Abbreviations

AECOPD: Acute exacerbation of COPD
HRV: Human rhinovirus
LOS: Length of stay
NPA: Nasopharyngeal aspirate
