# Supplementary material for: Molecular detection of respiratory pathogens and typing of human rhinovirus of adults hospitalized for exacerbation of asthma and chronic obstructive pulmonary disease
Source: Respir Res. 2019 Sep 13;20:210. doi: 10.1186/s12931-019-1181-0 (PMC6743175; doi:10.1186/s12931-019-1181-0)
Supplement: Supplementary file 1 — Additional file 1: Table S1. Length of hospital stay, readmissions and mortality in relations to types of human rhinovirus identified in the NPA in patients with acute exacerbation of COPD. Table S2. Length of hospital stay, readmissions and mortality in relations to types of human rhinovirus identified in the NPA in patients with acute exacerbation of asthma. Figure S1. NPA results of the COPD exacerbation cases in different months. Figure S2. NPA results of the asthma exacerbation cases in different months. (DOCX 145 kb) [file 12931_2019_1181_MOESM1_ESM.docx]

Molecular Detection of Respiratory Pathogens and typing of human rhinovirus of adults hospitalized for exacerbation of asthma and chronic obstructive pulmonary disease.

Fanny Wai-san KO^1^, Paul Kay-sheung CHAN^2^, Renee WY Chan^3^, Ka-Pang Chan^1^, April Ip^1^, Angela Kwok^2^, Jenny Chun-li Ngai^1^, So-Shan Ng^1^, Chan Tat On^1^, David Shu-cheong Hui^1^

^1^Department of Medicine and Therapeutics, The Chinese University of Hong Kong

^2^Department of Microbiology, The Chinese University of Hong Kong

^3^Department of Paediatrics, The Chinese University of Hong Kong

Supplementary Table 1. Length of hospital stay, readmissions and mortality in relations to types of human rhinovirus identified in the NPA in patients with acute exacerbation of COPD.

|  | HRV_A  n=15 | HRV_B  n=3 | HRV_C  n=8 | P-value |
| --- | --- | --- | --- | --- |
| Length of hospital stay (days) (Mean ± SD) | 13.1 ± 8.4 | 9.0 ± 8.6 | 6.9 ± 5.0 | 0.18 |
| 30-day Readmissions | 1 | 0 | 0 | 1.00 |
| 60-day Readmissions | 4 | 2 | 2 | 0.36 |
| In-hospital Mortality | 0 | 0 | 0 | NA |
| 30-Day Mortality | 0 | 0 | 0 | NA |
| 60-Day Mortality | 0 | 0 | 0 | NA |

HRV: Human rhinovirus; N/A: not applicable

Data are presented as mean±SD or number

Supplementary Table 2. Length of hospital stay, readmissions and mortality in relations to types of human rhinovirus identified in the NPA in patients with acute exacerbation of asthma.

|  | HRV_A  n=6 | HRV_B  n=1 | HRV_C  n=4 | P-value |
| --- | --- | --- | --- | --- |
| Length of hospital stay (days) (Mean ± SD) | 9.7 ± 6.9 | NA (only 1 case; length of stay was 3 days) | 7.7 ± 3.4 | 0.63 |
| 30-day Readmissions | 0 | 0 | 0 | NA |
| 60-day Readmissions | 0 | 0 | 1 | 0.38 |
| In-hospital Mortality | 0 | 0 | 0 | NA |
| 30-Day Mortality | 0 | 0 | 0 | NA |
| 60-Day Mortality | 0 | 0 | 0 | NA |

HRV: Human rhinovirus; N/A: not applicable

Data are presented as mean±SD or number


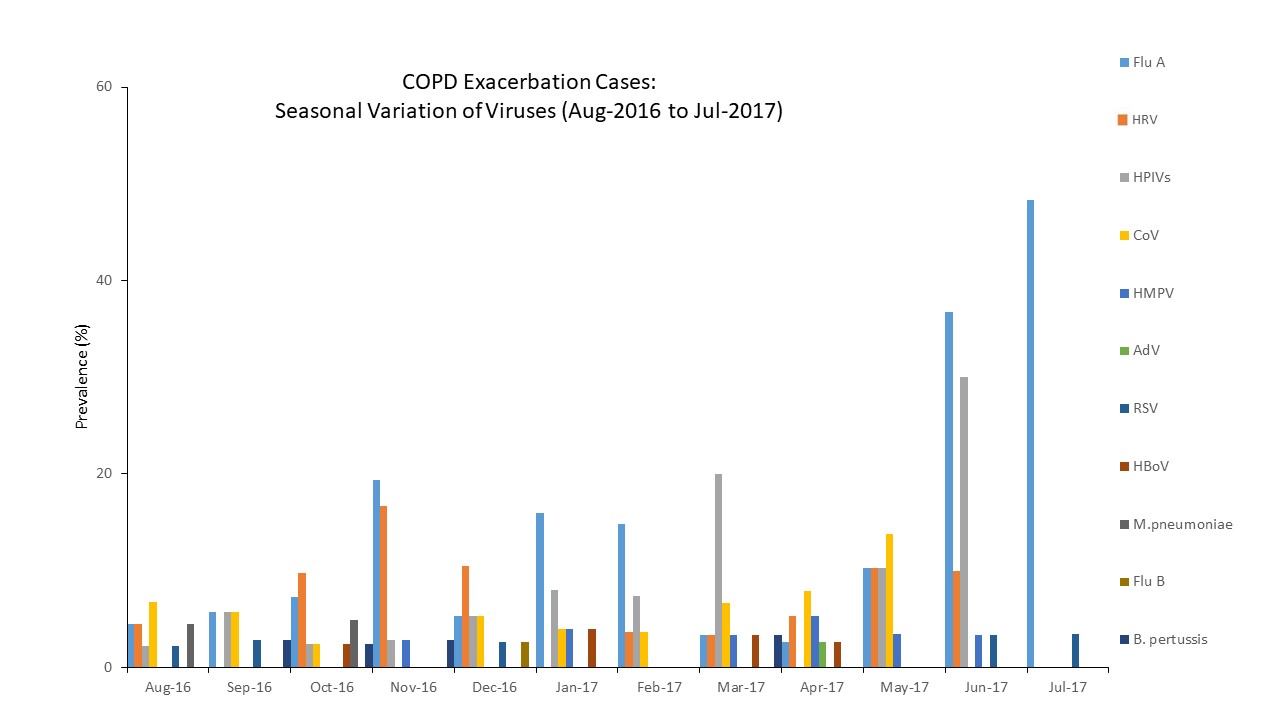


Supplementary Figure 1. NPA results of the COPD exacerbation cases in different months.
The bars represent the % of subjects with the microorganism identified over the total number of subjects recruited in that month.

ADV=adenovirus; B. Pertussis=Bordetella pertussis; CoV=Coronavirus; Flu A= influenza A; Flu B=influenza B; HBoV= Human Bocavirus; HMPV= Human metapneumovirus; HPIV=Human parainfluenza virus; M pneumoniae=*Mycoplasma pneumoniae*; HRVs= Human rhinovirus; RSV= Respiratory syncytial virus


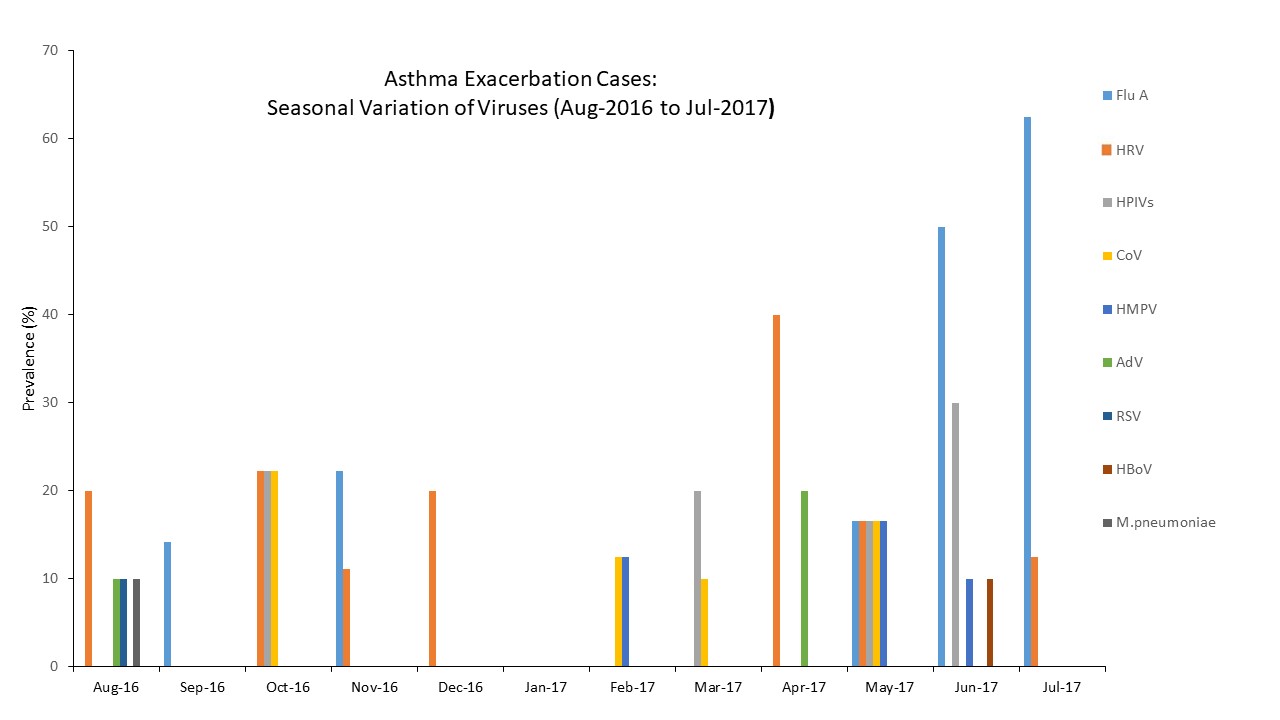


Supplementary Figure 2. NPA results of the asthma exacerbation cases in different months.
The bars represent the % of subjects with the microorganism identified over the total number of subjects recruited in that month.

ADV=adenovirus; CoV=Coronavirus; Flu A= influenza A; HMPV= Human metapneumovirus; HPIV=Human parainfluenza virus; M pneumoniae=*Mycoplasma pneumoniae*; HRVs= Human rhinovirus; RSV= Respiratory syncytial virus
